# Enhancement of nitrogen use efficiency through agronomic and molecular based approaches in cotton

**DOI:** 10.3389/fpls.2022.994306

**Published:** 2022-09-27

**Authors:** Muhammad Sohaib Chattha, Qurban Ali, Muhammad Haroon, Muhammad Junaid Afzal, Talha Javed, Sadam Hussain, Tahir Mahmood, Manoj K. Solanki, Aisha Umar, Waseem Abbas, Shanza Nasar, Lauren M. Schwartz-Lazaro, Lei Zhou

**Affiliations:** ^1^State Key Laboratory for Managing Biotic and Chemical Threats to the Quality and Safety of Agro-Products, Institute of Agro-Product Safety and Nutrition, Zhejiang Academy of Agricultural Sciences, Hangzhou, China; ^2^School of Plant, Environmental and Soil Sciences, Louisiana State University Agricultural Center, Baton Rouge, LA, United States; ^3^Laboratory of Integrated Management of Crop Diseases and Pests, Department of Plant Pathology, College of Plant Protection, Ministry of Education, Nanjing Agricultural University, Nanjing, China; ^4^National Key Laboratory of Crop Genetic Improvement and National Centre of Plant Gene Research (Wuhan), Huazhong Agricultural University, Wuhan, China; ^5^Department of Soil Science, University of Manitoba, Winnipeg, MB, Canada; ^6^College of Agriculture, Fujian Agriculture and Forestry University, Fuzhou, China; ^7^Department of Agronomy, University of Agriculture Faisalabad, Faisalabad, Pakistan; ^8^Department of Plant Breeding & Genetics, Pir Mehr Ali Shah Arid Agriculture University, Rawalpindi, Pakistan; ^9^Institute of Biology, Biotechnology and Environmental Protection, Faculty of Natural Sciences, University of Silesia in Katowice, Katowice, Poland; ^10^Institute of Botany, University of the Punjab, Lahore, Pakistan; ^11^Department of Botany, University of Gujrat Hafiz Hayat Campus, Gujrat, Pakistan

**Keywords:** nitrogen use efficiency, cotton, nitrogen metabolism, molecular approaches, physiological approach

## Abstract

Cotton is a major fiber crop grown worldwide. Nitrogen (N) is an essential nutrient for cotton production and supports efficient crop production. It is a crucial nutrient that is required more than any other. Nitrogen management is a daunting task for plants; thus, various strategies, individually and collectively, have been adopted to improve its efficacy. The negative environmental impacts of excessive N application on cotton production have become harmful to consumers and growers. The 4R’s of nutrient stewardship (right product, right rate, right time, and right place) is a newly developed agronomic practice that provides a solid foundation for achieving nitrogen use efficiency (NUE) in cotton production. Cropping systems are equally crucial for increasing production, profitability, environmental growth protection, and sustainability. This concept incorporates the right fertilizer source at the right rate, time, and place. In addition to agronomic practices, molecular approaches are equally important for improving cotton NUE. This could be achieved by increasing the efficacy of metabolic pathways at the cellular, organ, and structural levels and NUE-regulating enzymes and genes. This is a potential method to improve the role of N transporters in plants, resulting in better utilization and remobilization of N in cotton plants. Therefore, we suggest effective methods for accelerating NUE in cotton. This review aims to provide a detailed overview of agronomic and molecular approaches for improving NUE in cotton production, which benefits both the environment and growers.

## Introduction

### Source of nitrogen and plant soil interaction

Nitrogen (N) is the key component of plant chlorophyll, nucleic acids, and amino acids, and, compared to other elements, plants acquire N in large amounts from the soil (Shafreen et al., 2021). It is important for plant growth, leaf area, biomass, and crop yield (Kumar et al., 2020). Approximately 78% of the atmospheric N is dinitrogen gas (N_2_), which is converted into various forms of NH_4_^+^ and NO_3_^–^ by microorganisms. Undisturbed soil organic matter comprises almost 95% N (Walworth, 2013). From an agricultural perspective, certain N sources are major sources of available N in crop production. These are natural and organic N sources, some of which have been artificially developed. The conversion of N from one form to another greatly influences nitrogen use efficiency (NUE). At earlier stages, nitrate (NO_3_^–^) is essential, but it is not commonly used as a fertilizer alone, and the other forms are released into the atmosphere through the denitrification process. Although urea is the most widely used N fertilizer source, it is rapidly nitrified after its conversion to ammonium (NH_4_^+^) (Robinson et al., 2011). The application of urea to the soil results in NO_3_^–^ and NH_4_^+^. However, urea’s uptake process and the plants’ metabolic changes are not yet clear (Witte, 2011). Soil N availability in the soil is an indicator to examine the N efficiency in crop fields (Cui et al., 2008). Various field studies of N-labeled fertilizers have shown that N uptake is primarily obtained from the soil (Franco et al., 2011).

**FIGURE 1:**
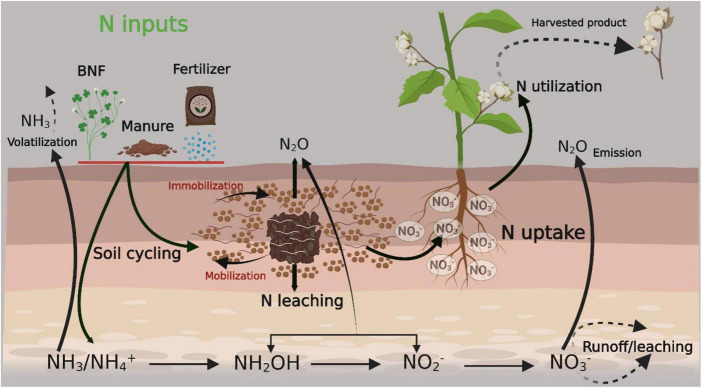
Summary of nitrogen sources and their conversion, availability to plant along with final harvested product and losses within/outside of soil. The figure created with Biorender (https://biorender.com/). BNF, biological nitrogen fixation.

Mineralization and bacterial N fixation are natural sources of available N in the soil (Iqbal et al., 2020c; Gu et al., 2022). NH_4_^+^ and NO_3_^–^ are the available forms of N for plant uptake. Nitrite (NO_2_^–^), nitrous oxide (NO), and atmospheric N are not readily available to plants unless they are converted into NH_4_^+^ and NO_3_^–^ (Ma et al., 2015; Su et al., 2022). Microorganisms also degrade N, which is naturally available in the soil. When plants die, they decompose and deposit N into the soil. Legume crops contribute more N than other field crops (Hocking and Reynolds, 2012). Legumes are grown during crop rotation, help fix atmospheric N, and deposit it into the uppermost layers of the soil. Animal bones and bone meals are also an important source of higher levels of N than chemical ones (Sharma and Bali, 2017). A summary of N sources and their conversion, availability to plants, final harvested product, and losses within and outside of the soil are presented in Figure 1.

One N and three hydrogens (H) form anhydrous ammonia (NH_3_), an easily available and relatively cheap N fertilizer. However, it is explosive, caustic, and toxic. The use of this fertilizer is strictly regulated in the United States [Occupational Safety and Health Administration (OSHA), 2017]. Urea is another source of N and is widely used in crop production. It is readily available in a granular form and is better to use in windy conditions. It is degraded into NH_4_^+^ and carbon dioxide (CO_2_). It is an excellent source of N with appropriate attention for better crop production. Ammonium nitrate (NH_4_NO_3_) is chemically composed of NO_3_^–^ and NH_4_^+^ cations. N uptake by plants is in the form of NH_4_^+^ and NO_3_^–^, and NH_4_NO_3_ is simply a mixture of both and is applied as a source of N. It has a low pH and is better for us in low wind conditions because of its structure (Kaiwen et al., 2015). One of the first N fertilizers produced 150 years ago was ammonium sulfate [(NH_4_)_2_SO_4_], which contains 21% N and 24% sulfur (S), which is most easiest to manage. (NH_4_)_2_SO_4_ is an important source of N and S, which are crucial for various plant functions, such as protein synthesis. (NH_4_)_2_SO_4_ is a well-known N fertilizer that lowers soil pH and enhances soil S. It is an excellent source of S in soil for better crop production (Khan et al., 2017b).

### What is nitrogen use efficiency and the common factors contributing to low nitrogen use efficiency?

Nitrogen, a structural component of proteins and DNA, is essential for life and considered the most important crop-yield limiting nutrient (Mueller et al., 2012). Therefore, most farmers rely on N fertilizers to increase crop yields and economic returns (McLellan et al., 2018). However, N is prone to different types of losses, including ammonia (NH_3_) volatilization, nitrate (NO_3_^–^) leaching, denitrification losses as dinitrogen (N_2_) gas emissions, and nitrous oxide (N_2_O) emissions, which lead to environmental pollution and contribute to climate change (Fowler et al., 2013). Recent studies have reported that the agricultural sector is a major source of N loss to the environment (Ahmar and Gruszka, 2022). The rapid increase in population and ever-increasing food demand will further increase the demand for N fertilizers in the future, which may consequently increase N losses unless significant improvements are made in the whole food production-consumption chain, and more appropriate N management strategies are developed (Chen et al., 2020a).

Nitrogen uptake, translocation, assimilation, and remobilization are complex processes referred to as nitrogen use efficiency (NUE). It also shows the extent of cotton lint and seed yield in response to N application (Shafreen et al., 2021). Cotton NUE results from N uptake efficiency (UpE) and utilization efficiency (UtE). Cotton NUE was represented by lint yield recorded after N application. UpE is defined as the total N uptake by plants recorded after N application, and UtE is the cotton yield ratio divided by total plant N. Under N deficiency, plant N UpE is more important than UtE (Witcombe et al., 2008). In addition, N is a mobile nutrient in the soil and is more prone to leaching than other soil nutrients (Shimono et al., 2009; Tariq et al., 2019). Leaching, runoff, and volatilization result in more N loss, and crop plants take up less N (Cavigelli et al., 2012). Therefore, sustainable crop production requires an improvement in NUE by reducing N loss (Khan et al., 2017a).

Improvement in NUE is associated with several agronomic practices, such as improved irrigation methods, improved fertilizer application considering the 4Rs, and using hybrids with greater potential yields and lower N inputs (Venterea et al., 2012). Efficient management, that is, N source, rate, time, and placement, increases NUE in cropping systems (Snyder et al., 2014). N inhibitors, split application of N, irrigation time, and correct placement method of fertilizer that considers soil and crop type improve overall NUE (Afridi et al., 2014; Halvorson et al., 2014). Recently, Tang et al. (2012a) reported an increase in N accumulation during the boll-setting stage with late N application (Tang et al., 2012a). Moreover, NUE is considered an important factor for fertilizer inputs to any agricultural system, as it maintains the N balance between inputs and outputs without any economic or environmental loss (McAllister et al., 2012). Application of N fertilizer at the first bloom stage is another way to improve NUE because cotton plants utilize N more efficiently for reproduction (Ali, 2015).

## Factors contributing to low nitrogen use efficiency

### Nitrogen losses

Half of the applied N is usually lost through NO_3_^–^ leaching, denitrification, or ammonia volatilization (Anthony and Armytage, 2008). Being negatively charged and highly soluble, NO_3_^–^ is prone to leaching when the soil becomes saturated after heavy rains or flood irrigation. Anoxic conditions develop and denitrifying microorganisms start using nitrate as an alternate e^–^acceptor, reducing NO_3_^–^ N to N_2_ (Shabbir et al., 2022). Weather conditions, irrigation patterns, and fertilizers also influence leaching (Follett, 2001; Riley et al., 2001). Bock and Kissel (1988) stated that alkaline soils resulting from NH_3_ volatilization lead to greater N loss because they have higher NH_4_^+^ concentrations on the soil surface. Various environmental factors, primarily higher temperature and wind speed, increase the risk of NH_3_ volatilization, which is a chemical process (Bock and Kissel, 1988). Soil characteristics, pH, cation exchange capacity (CEC), and moisture content also affect the volatilization rate (Jones, 2006).

### Temperature and soil characteristics

The nitrification process increases with an increase in soil temperature, resulting in N loss and consequently influencing NUE (Engel et al., 2011). The availability and loss of N in the soil depend on the soil type (Chen et al., 2010). The higher the CEC, the higher the soil buffering capacity and the rate of NH_4_^+^ absorption will be greater than the loss rate. Soils with lower anion capacity lose negatively charged molecules, such as NO_3_^–^ (Ferguson et al., 1984). Soil pH affects the activity of nitrifying and denitrifying bacteria (Ali et al., 2022a,c). The optimum pH for efficient N cycling is approximately 7 (Cameron et al., 2013). NUE is affected by the soil moisture content, which affects NO_3_^–^ leaching and the nitrification or denitrification rates (Darwish et al., 2006). NUE is positively correlated with the efficiency of irrigation systems (Bronson, 2008).

### Cropping systems, and C-N balance

The selection of cropping systems is crucial for balancing N input and output by improving N uptake and lowering the risk of N loss from the soil (Herridge et al., 2008). Several cropping systems tend to have a steady state of organic and inorganic soil N pools, with a slight change. Fast alteration in N pools in new soil management systems can influence the C-N balance as the soil organic matter remains constant. Overall, NUE in these cropping systems should incorporate changes in organic and inorganic soil N pools and N recovery efficiency (Lawlor, 2002). The C-N balance is an important factor unless adequate C is present, which improves the ability of plants to take up and utilize more N. Nitrogen levels can significantly affect C fixation and can be compromised (Castro et al., 1994). Photosynthetic rate and C level regulate N mineralization, uptake, assimilation, and immobilization. Hence, higher NUE can be attained through a higher photosynthetic rate (Lawlor, 2002).

### Nitrogen fertilizer type

Several N fertilizers are used without considering the soil type, crop genetics, and fertilizer chemistry, whereas an appropriate type of fertilizer reduces the percentage losses of N (Spicer, 2002). NH_3_ (82% N) is gradually converted to NO_3_^–^, with a minimal risk of N loss due to leaching or denitrification, while urea (46% N) rapidly transformed to NO_3_^–^. Wet or compact soils have serious denitrification issues, while coarse or no-till soils have higher leaching or volatilization. (NH_4_)_2_SO_4_ (21% N) applications have less or no volatilization losses. NH_4_NO_3_ (34% N) contains 50% NH_4_^+^ and 50% NO_3_^–^ when applied to the soil, and NH_4_^+^ N rapidly transforms into NO_3_^–^ N. NH_4_NO_3_ should not be used in soils subjected to leaching and denitrification; however, it is more suitable for surface applications (Nielsen, 2006).

### Nitrogen rates

The cotton crop requires almost 250–300 kg N ha^–1^ but utilizes only half to attain the maximum yield. Several studies have suggested that cotton uses N already available in the soil compared to applied N. On average, the cotton crop recuperates 33% of the applied N (Xu et al., 2012). In contrast, 25% of N remains in the soil until the maturity stage, with approximately 42% loss from the system (Tang et al., 2012b). Excessive N and N deficiency negatively affect plant growth and productivity (Liu et al., 2010; Yasmin et al., 2020). Moreover, extra N gradually leaches through underground water runoff, contaminating the groundwater with NO_3_^–^. Optimization of the N application rate depends on the soil type, climatic conditions, and several other soil and crop factors for better cotton production (Manghwar et al., 2021). However, the N rate for maximum economic yield depends on the N fertilizer cost and the commodity’s market price (Wajid et al., 2007). Owing to the complex chemical changes in N cycling that influence N loss, it is difficult to accurately predict the amount of N required. However, soil N availability, crop needs, and management practices should be considered to achieve greater NUE.

### Nitrogen timing

The application of N had a significant effect on the overall NUE. Generally, N is applied during three growth stages: pre-plant, first bloom, and peak bloom. The pre-planting application provides sufficient time for N conversion into plant-available forms. However, it is subject to higher risks of N losses, especially due to young seedlings’ lower requirement of N. N remains exposed to heavy rains for more than 60 days and is more prone to leaching losses (Halkier and Gershenzon, 2006; Ayaz et al., 2021). Moreover, nitrification and urease inhibitors are recommended for delaying nitrification and urea hydrolysis for fall-applied anhydrous ammonia and early applied urea, respectively (Lasisi et al., 2020).

## Approaches for improving nitrogen use efficiency

Improving NUE and guarding environmental quality are the two challenges cotton plant nutritionists face. NUE is described by several complicated interconnected factors that require close observation for improvement. Several field studies have revealed that site-specific N management tends to be more profitable and is considered sustainable. Several agronomic practices are also considered major factors influencing NUE.

### Spatial variation

Different pedological processes and management practices lead to spatial variability within the field (Bouma and Finke, 1993). These spatial variations can be horizontal along the horizons or vertically across depth. Variations in horizons have made it difficult to determine the boundaries of the soil types, while vertical variation is an indicator of the layers in soil classification (Mulla and McBratney, 2002; Kong et al., 2021b). Several studies have revealed that soil spatial variability is usually the main factor causing variation in soil properties and crop yield (Ping et al., 2005) and spatial and temporal variability in cotton yield (Ward and Cox, 2000). Cotton yield was found to be spatially correlated with a range of 23–40 m, fiber quality measurement was found to be spatially correlated with a range of 15–106 m, and cotton quality measurements were found to be spatially correlated (Johnson and Bradow, 2000; Bronson et al., 2003).

Ping et al. (2007) reported that soil NO_3_^–^ N highly depends on spatial variation. The spatial variability of soil properties suggests that site-specific cotton management is an appropriate option for improving cotton production with increased NUE (Ping et al., 2007). Site-specific management originated in efforts to adjust fertilizers to account for within-field variations in soil physical properties. Quantifying the spatial variability of soil properties and crop yield is important for decision-making in site-specific crop management because the spatial variability of soil and crop growth is the critical factor for determining variable-rate inputs of fertilizers and other chemicals. Improving the synchrony between demand and supply of N in the crop from all sources, that is, fertilizer application throughout the growing season, improves crop NUE (Cassman et al., 2003; Shankar et al., 2020; Ali et al., 2022b). However, site-specific N management is only possible following spatial variations. Recently, precision agriculture has provided an opportunity to address and measure spatial variability to promote sustainable agriculture and environmental stewardship (Ping et al., 2007; Ali Q. et al., 2021).

### 4R nutrient stewardship

An appropriate amount of N is required by crop plants for biomass production, and it helps restore soil organic carbon (C) levels (Lal, 2010). The best nutrient management practices (BNMPs) for N fertilizers play a key role in reducing nitrate leaching, lowering the risk of N_2_O emissions (a potent greenhouse gas), and improving overall NUE (Good and Beatty, 2011). Considering the weather and site-specific conditions when selection a suitable N fertilizer source can help improve NUE (Bruulsema et al., 2009; Ali M. et al., 2021). Intensive cropping systems meet the world’s need for food, fiber, and biofuel, which depend on better and sustainable crop production with improved NUE (Snyder et al., 2007; Solangi et al., 2021). Significant improvements in nutrient management practices may take several years to implement, as 4R practices are based on incremental progress and have an interim improvement/site-specific nature. However, implementing each management practice improves overall NUE and N_2_O emission mitigation (Wang et al., 2001).

**FIGURE 2:**
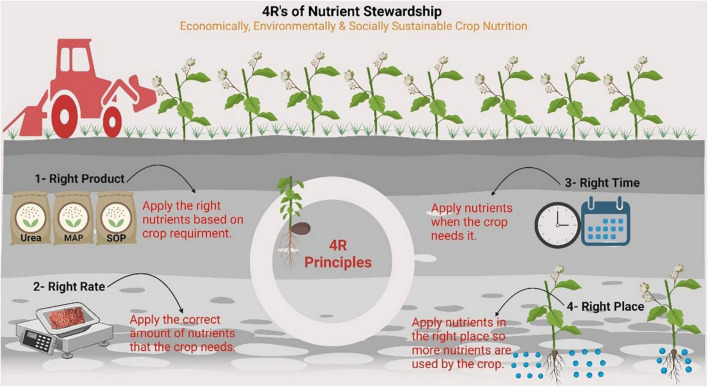
4Rs nutrient stewardship provides a framework to achieve cropping system goals, such as increased production, increased farmer profitability, enhanced environmental protection and improved sustainability. The figure created with Biorender (https://biorender.com/).

Nitrogen loss pathways include NH_3_ volatilization, denitrification, leaching, and runoff, leading to the formation of secondary aerosols, contamination of groundwater, and eutrophication, while N_2_O is emitted mainly from nitrification or denitrification processes (Ding et al., 2020; Manghwar et al., 2022). Thus, quantifying the different N loss pathways is important for developing proper N management strategies to improve overall NUE. One of the greatest factors leading to low N use efficiency and excess N losses in annual cropping systems is the mismatched timing of N availability with crop needs (Ranjan and Yadav, 2019). Recently, a 4R Nutrient Stewardship (4RNS) initiative has been developed and is being supported by the global fertilizer industry to achieve the basic economic (generation of more revenue per unit cropped area/input), social (production of better-quality food to meet global needs), and environmental elements (sustenance/improvement in soil quality and soil fertility, preservation of wildlife habitat, and biological diversity) of sustainability (Fixen, 2020). 4RNS is the adaptive management of mineral nutrients that has evolved through the refinement of interconnected practices and is site-specific (Bruulsema, 2018). The 4R practices that most clearly resulted in an improved NUE are presented in Figure 2.

### Right source, rate, time, and place

The key principle for selecting the right source is to ensure a balanced supply of nutrients, considering soil and crop plant characteristics, in addition to the rate, time, and placement method. Furthermore, we consider the synergism among nutrients and ensure the supply of nutrients in available forms to plants while selecting the right form. The use of enhanced N fertilizer sources with nitrification, urease, and dual inhibitors is a reliable means of reducing N losses and increasing NUE (Zhang et al., 2021). The greatest opportunities for increasing NUE were associated with lower rates of N fertilizer application. The key concept in selecting the correct rate is to assess the soil and crop needs in the source, time, and placement method. Rate-specific economics and fertilizer use efficiency (FUE) also play a key role in determining the correct rate. Better accounting for soil and residue N sources and targeting N rates for maximal N-use efficiency resulted in reduced overall N losses. To choose the appropriate fertilizer application time, split application of N fertilizers, especially for long-duration crops during the growing season, effectively reduces N losses with increased NUE. For example, the best time to apply N to late-sown cotton is at the appearance of the first flower. Afterward, cotton plants can utilize N much more efficiently in later stages (Zhang et al., 2021). Fertilizer placement can increase the efficiency of N fertilizer use by reducing NH_3_ emissions. The crop plant rooting pattern is the main deciding factor for the placement method, considering the source, rate, and time. It has also been shown that placement interacts with the tillage system, moisture, and temperature content to influence NUE. 4RNS is specifically built to achieve sustainability goals, including GHGs emission mitigation, particularly N_2_O, to reduce nutrient losses and produce more food per acre (Snyder et al., 2014). There are also opportunities to improve NUE associated with non-4R practices. Considering the effects of crop rotation on N availability, the impact of the tillage system, the use of tile drainage, and the inclusion of legumes in rotation are all important for improving NUE and developing 4R practices (Burton, 2018). Snyder et al. (2014) suggested that growers, crop advisors, researchers, policymakers, consumers, and the public could play a role in best management practices.

## Metabolic and molecular pathways of nitrogen use in plants and specifically in cotton

The study of plant metabolism is a key aim in plant research. An improved understanding of the metabolic activities will improve production and our understanding of the influence on the environment (Weißenborn and Walther, 2017; Yu et al., 2021). Several basic metabolic pathways, including the shikimic acid, mevalonate, amino acid, glutamine, proline, aspartate, carbohydrate, nitrogen (N), and lipid pathways (Rojas et al., 2014), have been studied in plants. All enzymatic steps are specific to the metabolic process and act on several families of associated molecules. As a result of metabolism, metabolic pathways produce several products accumulated in the same cell or different compartments or exported where needed. These molecules are channeled through metabolic pathways called metabolic flux. Other metabolites that regulate the primary and secondary metabolic pathways are also produced. Thus, it is difficult to understand the metabolic pathways (Farré et al., 2014).

Nitrogen metabolism is a basic and vital process for optimum plant growth, stress tolerance, and normal physiological processes (Zhong et al., 2017; Johnson et al., 2022). It is also an essential component of nucleic acids, chlorophyll, photosynthesis, RuBisCO, and some hormones (Liao et al., 2019). In the second half of the 20th century, nitrogenous fertilizers significantly augmented crops worldwide. However, excessive and limited application of nitrogenous fertilizers causes stagnation of cotton yields and reduces NUE. Moreover, external cues, such as biotic and abiotic stresses, also reduce crop yield by decreasing gas exchange and chlorophyll fluorescence triggered by increased resistance to CO_2_ diffusion and metabolic constraints (Zhong et al., 2017; Ihtisham et al., 2020). Exposure to drought alters plant metabolic activities and biological functions, which are responsible for restricted growth (Mahmood et al., 2019).

In cotton, the excessive use of nitrogenous fertilizers, very high costs, and external cues, such as biotic and abiotic stresses (Khan et al., 2017a; Sarraf et al., 2022) have become challenging tasks to improve NUE (Zhang et al., 2018). Furthermore, half of the applied nitrogenous fertilizers are not absorbed by plants and are leached, polluting groundwater reservoirs, which ultimately threatens ecosystems (Cameron et al., 2013). Iqbal et al. (2020b) studied four cotton genotypes and found that N concentration and different N-metabolic enzymes are important regulators of NUE. Additionally, 48 candidate genes involved in nitrogen metabolism were identified in wheat (Liu et al., 2020). A recent review by Ueda et al. (2017) emphasized the importance of N-responsive genes involved in the efficient uptake of N (Ueda et al., 2017). A recent study identified that the *TaPAP*, *TaUPS*, and *TaNMR* genes were differentially expressed in wheat with varying N levels. Expression studies revealed their roles as conserved N-metabolism genes. *TaNMR* has been identified as a novel gene in N metabolism (Li et al., 2019).

## Technical/metabolic pathway at the cellular, organ, and structural levels

Various studies on N metabolism have been conducted in wheat, rice, *Arabidopsis*, and cotton (Zhong et al., 2017; Vidal et al., 2020; The et al., 2021). N metabolism is a complex process that includes many physicochemical and biochemical processes, including N transportation, distribution, use, and reuse (Xing et al., 2018). Few studies have focused on N metabolism in cotton. However, even fewer studies describe the importance of N metabolism related to drought stress (Iqbal et al., 2020a), variation in N metabolism in response to NUE (Iqbal et al., 2020b), the effects of salt stress on N metabolism in roots and stems (Taliercio et al., 2010), the varying ratio of NH_4_^+^/NO_3_^–^ and its impact on N metabolism, higher N application, and increased N metabolism (Iqbal et al., 2020a) and recent advancements in N metabolism in cotton (Baslam et al., 2020).

**FIGURE 3:**
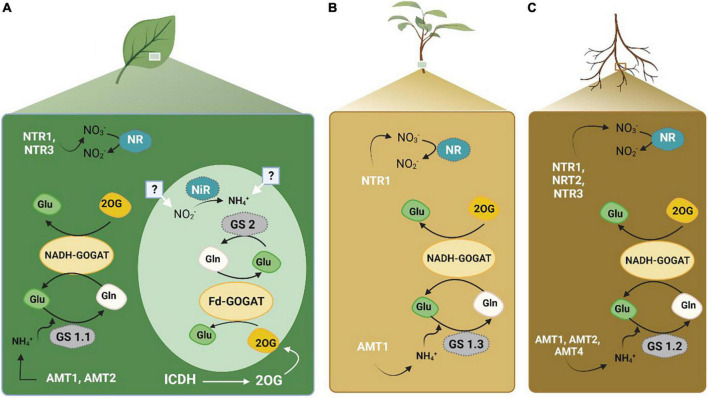
Nitrogen transporters and key enzymes involved in N metabolism and acquisition in plants. Panel **(A)** represents the activity in leaves. Panel **(B)** represents the activity in plant. Panel **(C)** represents the activity within the roots. The figure created with Biorender (https://biorender.com/).

Nitrogen metabolism (Figure 3) is one of the critical processes in plants that reduces nitrate and convert it into amino acids. It has proven to be influential in determining the NUE in cotton (Iqbal et al., 2020b). During this process, key enzymes including nitrate reductase (NR), nitrite reductase (NiR), glutamine synthetase (GS), glutamate dehydrogenase (GDH), glutamine synthase (GOGAT), asparagine synthetase (AS), and aspartate aminotransferase (AspAT), are used to make the N available to the plants in the form of amino acids (Xu and Zhou, 2004). These enzymes were assessed in citrus to determine biochemical markers of N status (Singh et al., 2018). The conversion of input N as a raw material to final product amino acids is mediated by a series of enzymes. NO_3_^–^ is converted to NH_4_^+^ by NR and NiR in the cytoplasm using one mole of NADPH or NADH, and NH_4_^+^ to glutamine in plastids using six moles of the reduced form of ferredoxin (Marcondes and Lemos, 2012; Ohyama et al., 2017). Initially, the enzyme GS, which consumes only one mole of ATP, assimilates the ammonium ion (NH_4_^+^) into Gln coupled with Glu. Furthermore, in plastids, 2 moles of ferredoxin are used by GOGAT to convert Gln into an organic acid, 2 oxoglutarate (2-OG) (Crawford, 2000). As a result of the transfer of Glu into organic acids, several amino acids (AA) are produced via transaminases. Previously, NH_4_^+^ was viewed as a factor responsible for the assimilation of Glu by GDH. However, recent research has validated the GS/GOGAT cycle as the principal route of ammonium assimilation in plants (Crawford, 2000; Crawford and Forde, 2002).

Previously, cotton was an orphan crop; many studies have been conducted to increase the NUE by focusing on the N concentrations and morphological and biochemical traits, but not much has been done on N metabolism and related enzymes, such as NR, NiR, GS, GOGAT, and GDH, which carry out the whole process from N uptake to use (Abenavoli et al., 2016; Luo and Zhou, 2019). Furthermore, N-containing compounds, such as amino acids and proteins, are key partners of N metabolism processes, including assimilation and metabolism, which determine genotypic responses to N supply (Quan et al., 2017). Therefore, these enzymes and N metabolism are considered the most vital biochemical factors for improving NUE in cotton (Xu et al., 2012). Another study was conducted to determine the contrasting NUE of six cotton genotypes. Biochemical and morpho-physiological traits such as N metabolic enzymes, shoot dry weight, and root traits were mostly affected in response to varying nitrate concentrations. NUE positively correlated with improved N uptake efficiency (Iqbal et al., 2020d).

In another study on cotton (Iqbal et al., 2020b), a close association between N uptake and utilization efficiency was demonstrated by applying various N doses. Because it is challenging to improve NUE by lowering N supply and selecting N efficient cotton genotypes, uptake, utilization, and remobilization of available N. Based on a contrasting N metabolic study, N uptake efficiency (NUpE), and N utilization efficiency (NUtE), CCRI-69 and XLZ-30 showed efficient NUE (Iqbal et al., 2020b). The N concentration and N-metabolizing enzymes were attributed as important traits that confer high NUpE. After applying higher N concentrations, shoot and root NR, GOGAT, and GDH enzyme activities increased. However, different genotypes exhibited contrasting behaviors (Iqbal et al., 2020b).

## Nitrogen transporters and role in nitrogen use efficiency

**FIGURE 4:**
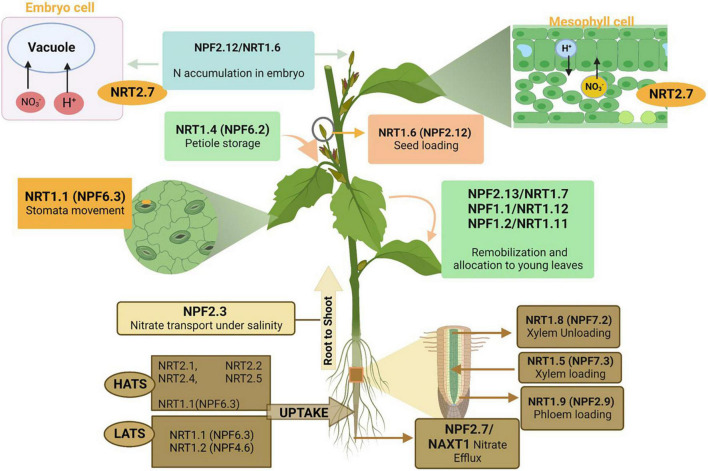
Roles of different nitrogen transporters in nitrate uptake and efflux from the soil, transportation from roots to shoots, allocation and assimilation in plant leaves, and seed development. As mentioned in the above section, these nitrogen transporters are linked to different families. The figure created with Biorender (https://biorender.com/).

Plants absorb N in the form of nitrate at the root level by four nitrate transporter families: the nitrate peptide family NPF (previously known as NRT1/PTR), nitrate transporter 2 family NRT2/NNP (Nitrate-Nitrite Porter), chloride channel/transporter family (CLC-1), and slow anion associated channel homolog (SLAC1/SLAH) family (Iqbal et al., 2020d) (Figure 4). However, according to recent literature (Fan et al., 2017), the NRT1 (NPF) and NRT2 families are considered the main nitrate transporters for the uptake and transfer of nitrate in plant roots (Fan et al., 2017). NPF and NRT2 families are mainly responsible for low-affinity transport systems (LATS) and high-affinity transport systems (HATS), respectively (Williams and Miller, 2001; Tsay et al., 2007), whereas NRT1⋅1 (NPF6.3) and NRT1.3 (NPF6.4) (Morère-Le Paven et al., 2011) are responsible for both LATS and HATS (Liu et al., 1999). However, at high nitrate concentrations (>1 mM), it responds toward LATS (Raddatz et al., 2020). The NRT1 (NPF) nitrate transporters in other crops and transporters that behave differentially based on the varying nitrate concentration has been discussed in detail (Segonzac et al., 2007; Morère-Le Paven et al., 2011; Hawkesford, 2012; Hu et al., 2015; Chen et al., 2016).

In plants, multiple nitrate transporters (Table 1) perform NO_3_^–^ uptake. Both NRT1 (NPF) and NRT2 transporter families affect plant growth and seed development because of the differences in NO_3_^–^ uptake efficiency (Wang et al., 2020). NRT1 (NPF) was identified in Arabidopsis (Tsay et al., 1993) and is part of the NPF family (Léran et al., 2014). The NPF family includes 53 genes in Arabidopsis, 139 were identified in higher plants, and 93 genes in rice. These are further subdivided into 8–10 families as reviewed by Iqbal et al. (2020d); NPF6.3 (NRT1.1) and NPF4.6 (NRT1.2) are responsible for NO_3_^–^ uptake in roots (Iqbal et al., 2020d). NRT1.1 (NPF6.3) is the first and most studied NO_3_^–^ transporter in plants (Tsay et al., 1993). In the NRT1 (NPF) family, Arabidopsis includes 53 genes, of which 51 show differential expression patterns in the whole plant (Tsay et al., 2007). The NRT1 (NPF) family functions as NO_3_^–^ transporters and a diverse range of substrates, including abscisic acid, nitrite, amino acids, peptides, chloride, glucosinolates, gibberellins, auxin, and jasmonoyl-isoleucine (Krouk et al., 2010; Kiba et al., 2012; Nour-Eldin et al., 2012; Saito et al., 2015; David et al., 2016; Tal et al., 2016).

**TABLE 1:** Various nitrogen transporters involved in different functions of nitrate uptake, utilization, and remobilization.

Sr. no.	N transporters	Function/s	References
(1)	NPF2.7/NAXT1	Root efflux	O’Brien et al., 2016
(2)	AMT1;1, AMT2;1, NPF6.2/NRT1.4, LHT1	Leaf import	Noguero and Lacombe, 2016
(3)	NPF2.12/NRT1.6, UmamiT11, UmamiT14	Phloem unloading, and senescing	Noguero and Lacombe, 2016
(4)	NRT2.7	Seed vacuole	O’Brien et al., 2016
(5)	NPF7.2/NRT1.8, NPF6.2/NRT1.4, NPF1.2/NRT1.11, NPF1.1/NRT1.12, AAP6, AAP2	Xylem and phloem transfer	Fan et al., 2017
(6)	CLCa, AVT3a	Leaf vacuole	Tegeder and Masclaux-Daubresse, 2018
(7)	DiT2	Chloroplast	Tegeder and Masclaux-Daubresse, 2018
(8)	NPF2.13/NRT1.7, NPF1.2/NRT1.11, NPF1.1/NRT1.12, NRT2.4 NRT2.5, UmamiT18 AAP8	Leaf export and phloem loading	Zhang et al., 2018
(9)	NPF5.5, UmamiT28, UmamiT29, CAT6, AAP8, AAP1	Loading to seed	Tegeder and Masclaux-Daubresse, 2018
(10)	AMT1.1, AMT1.2, AMT1.3, AMT1.5, NRT2.1, NRT2.2, NRT2.4, NRT2.5, NPF6.3/NRT1.1, NPF4.6/NRT1.2, AAP1, AAP5, LTH1, LTH6, ProT2, ANR1	Nitrogen uptake	Tegeder and Masclaux-Daubresse, 2018
(11)	NPF7.3/NRT1.5, NPF2.3, UmamiT14, UmamiT18, UPS1-1, UPS1-2	Movement from root nodule to xylem	Tegeder and Masclaux-Daubresse, 2018
(12)	NPF7.2/NRT1.8, NPF2.9/NRT1.9	Root reimport	Fiaz et al., 2021

Eight NRT2 transporters that respond to HATS have been identified in different plants (Von Wittgenstein et al., 2014). Although not many nitrate transporters have been identified in, we present the transporters from other plants such as Arabidopsis and rice. In Arabidopsis, several NRT2 transporters have been identified; four of them, including NRT2.1, NRT2.2, NRT2.4, and NRT2.5, function in nitrate influx and play a role in 95% nitrate uptake under low NO_3_^–^ concentrations. However, NRT2.1 and NRT2.2 are the principal members of the NRT2 family for nitrate uptake (Lezhneva et al., 2014). Although NRT2.1 is a member of HATS but works efficiently under low nitrate availability (Li et al., 2007), its HATS activity is reduced, and plant growth is affected by low NO_3_^–^ (Skopelitis et al., 2006) as demonstrated using mutants (Carvalho et al., 2003). However, compared to NRT2.1, NRT2.2 showed less expression for the uptake of NO_3_^–^ (Suzuki and Knaff, 2005). Moreover, NRT2.4 and NRT2.5 are also responsible for NO_3_^–^ uptake, but NRT2.4 was identified as a high-affinity transporter, as its mutant showed a reduction in NO_3_^–^ uptake upon 0.025 mM nitrate (Kiba et al., 2012). Overall, NO_3_^–^ acquisition depends on the specificity of NO_3_^–^ transporters because NRT2.4 and NRT2.5 absorb NO_3_^–^ from soil root hairs while NRT2.1 and NRT2.2 transport it from the apoplast to the apoplast cortex and endodermis (Suzuki and Knaff, 2005). In response to long-term starvation, NRT2.5 absorb NO_3_^–^ efficiently from shoots and roots of adult plants, as its expression is increased along with several other NRT2 transporters (Lezhneva et al., 2014). In addition, NAR2(NRT3) from the NRT3 family is another transporter that develops a coupling relationship with NRT2 to transporters to NO_3_^–^ in plants (Li et al., 2007). Many NRT2 transporters have been identified and studied in other plants, including *Chlamydomonas reinhardtii* (Zhou et al., 2000), barley (Marschner, 2011), and rice (Chen et al., 2016). These NRT transporters can be manipulated to improve NUE.

NH_4_^+^ uptake is carried out by ammonium transporters (AMTS) and other regulators such as cation channels or aquaporins (Glass et al., 2002). However, the overexpression of genes responsible for ammonium transporters has not yet been successful (Meister et al., 2014). Because of the excess availability of NH_4_^+^ in cells, it becomes toxic, a possible hindrance to targeting NH_4_^+^ transporters to improve N uptake (Bittsánszky et al., 2015). In addition to inorganic N acquisition, plants absorb organic nitrate in amino acids (AA) (Näsholm et al., 2009). Various root transporters, including AAP1 and AAP5, proline transporter ProT2, and lysine-histidine-type transporters LHT1 and LHT6, are responsible for the uptake of amino acids (The et al., 2021). However, this is only possible in fields that rely on manure or compost (Enggrob et al., 2019).

After NO_3_^–^ uptake, it is assimilated in different parts of the shoot and loaded into the xylem vessels of roots using several transporters such as NRT1(NPF)/PTR and NRT (O’Brien et al., 2016). Upon assimilation, it is converted into AA (Meyer and Stitt, 2001). More assimilation takes place in shoots that in roots (O’Brien et al., 2016), using the same process discussed earlier. Different NO_3_^–^ transporters have been shown to increase N assimilation in plants. According to a recent review (O’Brien et al., 2016), NRT1(NPF)/PTR and NRT2 members are expressed in the xylem and phloem. In Arabidopsis, NPF7.3 (NRT1.5) (Meng et al., 2016), NPF7.2 (NRT1.8) (Tong et al., 2005), and NPF2.9 (NRT1.9) (Wang and Tsay, 2011) play roles in influx/efflux, removal of NO_3_^–^ from the xylem, and loading of NO_3_^–^ into the root phloem, respectively. Besides these, many other genes are also responsible for the better uptake, assimilation, and remobilization of NO_3_^–^ from roots to shoots (Fan et al., 2017).

According to the cited literature (Fang et al., 2013; Fan et al., 2016a; Feng et al., 2017; Wang Y. Y. et al., 2018), only NRT(NPF) transporters have been overexpressed in both leaves and roots to improve the NUE (The et al., 2021). However, it is yet to be determined whether overexpression of these NRT2 transporters in roots is sufficient to improve NUE. In the latest research on Arabidopsis, rice, and tobacco, overexpression of the hyperactive chimeric NO_3_^–^ transporter *AtNC4N* in the phloem of old leaves increased N uptake and improved NUE under low N levels. Another study showed that *the OsNRT1.1A* (*OsNPF6.3*) NO_3_^–^ transporter gene is involved in improving NUE, flowering, high yield, and early maturation in rice. Many other N transporters, such as NRT1.1B (NPF6.3B), NRT2.1, NRT2.3a, NRT2.3, NRT2.3b, PTR9, AMT1.1, and qNGR9, have been shown to increase NUE under high and low N concentrations (Fang et al., 2013; Ranathunge et al., 2014; Sun et al., 2014; Fu et al., 2015; Hu et al., 2015; Chen et al., 2016, 2017, 2020b; Fan et al., 2016a,b). Furthermore, several other genes in different plants have been shown to improve plant growth and NUE, including the nitrate transporter *OsNPF4.5* (Sun et al., 2014), *NAC42-*activated nitrate transporter (Tang et al., 2019), and nitrate reductase gene *OsNR2* (Gao et al., 2019).

## Molecular and signaling pathways involved in nitrogen use efficiency

How the nitrate signaling and gene expression networks can be used to improve NUE in plants are not yet completely understood. Additionally, researchers have attempted to increase NUE by modulating the expression of key genes involved in NO_3_^–^ uptake, assimilation, and remobilization in various plants. However, no significant success has been recorded (McAllister et al., 2012). Thus, success cannot be achieved until all other processes, including uptake, transport, assimilation, and remobilization, are understood. To improve NUE, it is also necessary to understand these processes and how they coordinate the expression of genes involved in the NO_3_^–^ response. Apart from the N source, NO_3_^–^ also modulates gene expression and plays a role in various developmental processes, including seed germination, shoot development, flowering, and root architecture (O’Brien et al., 2016; Lin and Tsay, 2017; Liu et al., 2017; Kant, 2018; Wang Y. Y. et al., 2018).

NO_3_^–^ induces a primary NO_3_^–^ response that regulates the transcriptional response without requiring *de novo* protein synthesis (Gowri et al., 1992). As a result of this rapid response, gene expression can be induced within minutes, reaching a peak at approximately 30 min. However, NO_3_^–^ concentration determines the induction levels of PNR genes (Hu et al., 2009). Transcriptome analysis revealed the importance of NO_3_^–^ in gene expression, as it controls 10% of the genome (Bouguyon et al., 2012). Various PNR gene families have been studied in plants, such as NRT1(NPF), NRT2, NIR, NIA1, NIA2, and other genes responsible for different metabolic processes, including the trehalose-6-P metabolism pentose phosphate pathway and glycolysis (Scheible et al., 1997). Several main NO_3_^–^ signaling pathways, including transcription factors, peptides and proteins, kinases, NO_3_^–^ transporters, and calcium signaling. Because cotton has not been well studied regarding NO_3_^–^ molecular signaling pathways, we focused on these pathways in other crops to understand the potential mechanism in cotton.

NRT1.1 (NPF6.3) is involved in the uptake of NO_3_^–^. Two NO_3_^–^ transporters, NRT1.1 (NPF6.3) and NRT2.1, are responsible for NO_3_^–^ signaling and sensing. NRT1.1 (NPF6.3) also regulates the expression of NRT2.1 (Munþos et al., 2004). In the case of short-term and long-term NO_3_^–^ supply, NRT1.1 (NPF6.3) upregulates NRT2.1, which involves the feedback repression of NRT2.1 (Bouguyon et al., 2015). Based on its dual-affinity property, it can switch between high- and low-level NO_3_^–^ responses (Hu et al., 2009), which is caused by the phosphorylation status of the T101 residue (Hu et al., 2009). Furthermore, NO_3_^–^ signaling is differentially regulated by the interaction of two CALCINEURIN B-LIKE (CBL)-INTERACTION PROTEIN KINASES (CIPKs) with NRT1.1 (NPF6.3), and CIPK8 and CIPK23 engage in low- and high-affinity responses, respectively (Ho et al., 2009). Except for PNR regulation, NRT1.1 (NPF6.3) is also involved in the root during the aging process. NRT1.1 (NPF6.3), which plays a role in sensing NO_3_^–^ concentration as root growth depends on NO_3_^–^ sensing; at high and low NO_3_^–^ concentrations, lateral root growth is promoted and inhibited, respectively. At high NO_3_^–^ concentrations, NRT1.1 (NPF6.3) upregulates Arabidopsis NO_3_^–^ regulated 1 (ANR1) for root proliferation and lateral root growth development (Remans et al., 2006). While At low NO_3_^–^ concentrations, NRT1.1 (NPF6.3) controls auxin levels and meristem activation for the repression of lateral root development (Mounier et al., 2014). An additional NO_3_^–^ sensing system is also present, as the abolishment of NRT1.1 (NPF6.3) does not disrupt the PNR system, but its exact function in NO_3_^–^ signaling is not understood (Sun et al., 2017).

After NO_3_^–^ sensing by NRT1.1 (NPF6.3), the next step is to transmit the NO_3_^–^ signals to the nucleus, which magnifies the cytosolic regulators. Calcium and various transcription factors are the main role players in signal transduction. Three CIPKs (CPK10, CPK30, and CPK32) and their partner CBL indicated that calcium also functions in NO_3_^–^ signaling (Krouk, 2017). Calcium acts as a secondary messenger in the NO_3_^–^ signaling pathway (Liu et al., 2017), responsible for changes in gene expression (Medici and Krouk, 2014). The first time, this study was conducted 30 years ago on barley and maize, where it showed that NO_3_^–^ responsive genes show varying expression as a result of EGTA or LaCl3 pretreatment (Sakakibara et al., 1997). Furthermore, its function as a NO_3_^–^ signaling pathway has been investigated in various studies (Riveras et al., 2015). Moreover, many other transcription factors (TFs), like NLP7, TCP20, LBD37, LBD38, LBD39, CIPK8, SLP9, TGA1, TGA4, CIPK23, bZIP1, BT1, and BT2 have been identified that interact with nitrate responsive genes, carry out the function of NO_3_^–^ response, and improve NUE (Wang Y. Y. et al., 2018).

Based on recent bioinformatics analyses, BT1 and BT2 have proven to be great assets for improving NUE. BT1 was identified as the closest homolog of BT2. BT1 and BT2 improve NUE by repressing the expression of the nitrate transporters NRT2.1 and NRT2.4, which reduces NO_3_^–^ uptake (Araus et al., 2016). Both TFs are involved in plant growth. However, it is yet to be determined whether BT1 and BT2 target multiple genes under N-sufficient and N-deficient conditions. The RWP-PK TFs family includes NLP7 TF. In a genome-wide analysis, NLP7 was identified as a player in modulating the expression of various genes involved in NO_3_^–^ signaling, uptake, and assimilation (Marchive et al., 2013). Similarly, SPLN also regulates the expression of different NO_3_^–^ transporter genes, including *NPF6.3* (*NRT1.1*), *NRT 2.1, NRT 2.2, NIA1, NIA2*, and *NIR* (Krouk et al., 2010).

## Cross talk on source/sink relationship and nitrogen use efficiency

**FIGURE 5:**
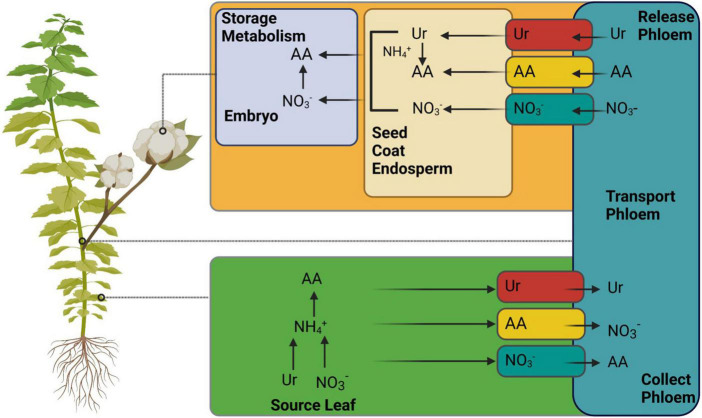
Illustrates the mechanism of nitrogen remobilization from source (leaf) to sink (seed). Various known and unknown transporters are involved in portioning, as mentioned in Tegeder and Masclaux-Daubresse (2018). AA, amino acids. The figure created with Biorender (https://biorender.com/).

Generally, N is recycled and remobilized in source leaves after uptake by the roots. It is delivered to the sinks via the phloem as amino acids, NO_3_^–^, and ureides (Figure 5) (Tegeder and Masclaux-Daubresse, 2018). However, different agronomic characteristics, such as NUE, grain filling, and yield, depend on better N remobilization in the source and its allocation to sink organs. The removal of N is initiated mainly during leaf senescence (The et al., 2021). In plants, senescence is a developmental process that regulates the nutrient requirements. Therefore, leaf senescence is also linked to the source/sink relationship (Guiboileau et al., 2010). In addition to the natural aging process, many other factors, such as nutritional starvation, pathogen infections, C-N ratio, photosynthetic activity, photoperiod, C accumulation, and various other cues, can initiate senescence. The limited availability of N also increases leaf senescence earlier than the sufficient availability in sunflowers (Bieker and Zentgraf, 2013).

In addition to N, some environmental factors, such as light quality and quantity and reactive oxygen species (ROS) (Zimmermann et al., 2006; Kong et al., 2021a), induce leaf senescence, which ultimately regulates nutrient mobility from source to sink organs where needed. Different sink organs and developmental processes require N. During anthesis, it varies according to the genotype. Varying concentrations of N are responsible for the differential uptake of N among different Arabidopsis accessions. Upon application of High N concentrations, most of the N is remobilized to the seeds, whereas at low N concentrations, it is allocated to rosette leaves (Masclaux-Daubresse and Chardon, 2011). During seed filling and flowering, the uptake of N is arrested in response to a reduction in HATS and HATS + LATS activities (Masclaux-Daubresse et al., 2010). Here, we will focus on natural-induced senescence accompanied by the movement of nitrogenous nutrients from source leaves to sink organs and various efforts to increase NUE in response to the source and sink relationship.

Generally, due to autophagy, proteins, organelles, and cytosolic macromolecules are degraded during leaf senescence, and N is translocated toward sink organs (Chen et al., 2019). Associated cytoplasmic components are dismantled and degraded during autophagy, which plays an important role in regulating and remobilizing nutrients from source to sink organs (Tegeder and Masclaux-Daubresse, 2018). *ATGs* genes are responsible for this autophagy process during leaf senescence, and are upregulated during autophagy (Chung et al., 2010; Breeze et al., 2011). Autophagy-related procedures can degrade the chloroplast and the most abundant protein, Rubisco, accounting for 80% of cellular N (Wada et al., 2009). This is further reinforced by the fact that leaf senescence is initially observed because of chloroplast dismantling and degradation. According to the cited literature (Martínez et al., 2008), enlisted protease enzymes, including chloroplastic DegP, FstH, and CND41 are responsible for the degradation of proteins D1 and Rubisco, respectively. Details regarding protein degradation mechanisms are provided by various studies (Tegeder and Masclaux-Daubresse, 2018; Gill et al., 2021). One of the efficient methods, the “apparent remobilization” method, which works based on N^15^ labeling, is used to orchestrate the amount of N present in different developmental stages of plants (Gallais et al., 2006).

Seeds are the major sink organs during N remobilization, which depends on different factors: strength of the seed sink, translocation processes in leaves, stems, and reproductive organs, and efficiency of phloem pathways (Manghwar et al., 2022). Based on acquired knowledge in different plants, it is well known that asparagine and glutamine are the major translocated amino acids from source to sink organs. Their concentrations also increased during leaf senescence. The amino acid permease (AAP) family is a candidate gene for improving the phloem loading efficiency. Different genes, including *AAP4, AAP5, BnAAP1*, and *BnAAP2*, have been implicated in phloem loading (Fischer et al., 2002; Koch et al., 2003; Chaffei et al., 2004; Tilsner et al., 2005). During leaf senescence, N remobilization and assimilation can improve the NUE of plants. Both GS1 and GS2 proved influential in improving NUE. Further improvement of N remobilization depends on the severity and severity of leaf senescence activity (Diaz et al., 2008).

There is a long list of protease genes responsible for protein degradation and N remobilization. Among these genes, *SAG12* is a widely studied enzyme that catalyzes the cysteine protein in leaves. In addition, its homologs are also present in other crops and are responsible for N remobilization and improvement of NUE in oilseed and tobacco (Chung et al., 2010). Furthermore, glutamate dehydrogenase (*GDH*) is also important for improving NUE, as it functions to remobilize N (Lea and Miflin, 2011). However, overexpression of *GDH* did not result in N remobilization as in the case of tomato *Slgdh-NAD: B1* in tobacco, *GDH* from *Sclerotinia sclerotiorum* and *Magnaporthe grisea* in rice (Purnell et al., 2005), and *Nicotiana plumbaginifolia GDHA* and *GDHB* in *Nicotiana tabacum* (Tercé-Laforgue et al., 2013). In contrast it improved NUE in other plants, such as overexpression of *EcgdhA* from *Escherichia coli* in tobacco and maize (Ameziane et al., 2000). In addition, overexpression of *AngdhA* from *Aspergillus nidulans* in potato (Egami et al., 2012) and fungal *GDH* from *Cylindrocarpon ehrenbergii* (*CeGDH*) in rice (Zhou et al., 2015) augmented NUE. Autophagy respondent genes such as AtATG8 in Arabidopsis, OsATG8b, OsATG8a, and OsATG8c in rice also proved valuable in improving NUE under sufficient conditions of N supply (Gallais et al., 2006; Zimmermann et al., 2006).

Other factors, such as N remobilization in the form of inorganic or ionic N, or combined with organic molecules, also play a differential role in improving NUE. Different studies have reinforced the significant role of inorganic N in N remobilization toward sink organs, but it also depends on the availability of sufficient N inputs (Masclaux-Daubresse et al., 2010). Various studies on NO_3_^–^ transporters, including NRT2.5, NRT1.6 (NPF2.12), NRT1.5 (NPF7.3), and AMT1.5, could improve NO_3_^–^ remobilization from source to sink organs, which ultimately also increases NUE (Wu et al., 2014; Havé et al., 2017). Another study was conducted on the NO_3_^–^ transporter NRT1.7 (NPF2.13). They manipulated its remobilization, which improved NUE and increased plant growth, as it was involved in the meditation of stored NO_3_^–^ from the source to sink organs (Chen et al., 2020c).

In addition to molecular-level studies, some common agronomic practices are also essential for better source-to-sink relationships in plants. Research on efficient and inefficient cotton genotypes, CCRI-69 and XLZ-30, showed that moderate to high N applications improve the remobilization of the source-to-sink relationship, which ultimately increases the NUE and yield (Iqbal et al., 2020c). Furthermore, different foliar applications also proved beneficial. Under treatment with different N foliar applications, such as NO_3_^–^ and urea, winter wheat plants showed increased grain filling and source-to-sink relationships of N remobilization, as (Lyu et al., 2020; Liang et al., 2022). However, many N studies have been conducted on plants other than cotton plants. Currently, there is a dire need to study cotton plants to improve the NUE and source-to-sink relationship for better remobilization of N by employing molecular and agronomic studies.

Crop plants largely absorb N in the form of nitrate (NO_3_^–^) (Robinson et al., 2011; Manghwar et al., 2022), or ammonium (NH_4_^+^) when growing in acidic soils (Robinson et al., 2011; Wang L. et al., 2018). After uptake from the soil, plant roots assimilate NH_4_^+^ to avoid the toxicity of free ammonium, which would eliminate the transmembrane proton gradients pivotal for respiratory electron transport. Unlike ammonium, a large concentration of nitrate ions are assimilated in plant tissues and then translocated to plant shoots for assimilation. It is well established that N rates and sources significantly affect plant growth and developmental process (Irshad et al., 2008; Lu et al., 2009). Therefore, selecting the appropriate rate and method based on plant species, growth stage, soil, and environmental conditions is necessary to maximize plant growth and NUE (Marschner, 2011; Niu et al., 2011; Mendoza-Villarreal et al., 2015). Other farm management practices, including genotype selection, also significantly improved NUE. Genotype development and screening have been reported to be imperative for improving the uptake and utilization of N (Iqbal et al., 2020b).

In a recent study, Iqbal et al. (2020a) evaluated the performance of N-efficient and N-inefficient cotton genotypes supplied with nitrate and ammonium-N. They reported increased N uptake and utilization efficiency, better plant growth, chlorophyll content, and gas exchange in the N-efficient genotype when supplied with nitrate-N (Iqbal et al., 2020a). Improved root traits in nitrate-fed plants, including length, dry weight, and surface area, have been reported previously (Schortemeyer and Feil, 1996). Another study also reported better seedling growth in cotton when fed nitrate N than ammonium-fed seedlings, mainly due to better photosynthesis under increased translocation of nitrate to the photosynthesis system (Irshad et al., 2008). Ammonium application is also not recommended, as it reduces the uptake of potassium and cations to retard stomatal function and inhibit osmotic regulation, respectively (Lopes and Araus, 2006). Similarly, low NUE in cotton under ammonium application has also been attributed to inhibited ammonium metabolism and reduced protein synthesis (Pessarakli and Tucker, 1985). Chen et al. (2020a) studied different N application rates under field conditions. They reported that a moderate N application rate (240 kg ha^–1^) significantly increased seedling growth and cotton yield, thereby promoting NUE (Chen et al., 2020a). Similarly, in a recent study, Wang et al. (2021) reported that N application at 250 kg ha^–1^ considerably increased the N uptake and NUE in cotton (Wang et al., 2021).

## Increasing plant nitrogen utilization and remobilization efficiency in plants

Nitrogen application rate, time of application, the N source, and other agronomic practices affect crop yield, N uptake, and its movement and metabolism within the plant, depending on the plant species (Ali, 2015; Ali et al., 2022a). Xu et al. (2012) studied the effects of different planting densities and reported that increasing planting densities in cotton enhanced N accumulation in plant parts (Xu et al., 2012). Similarly, Yao et al. (2015) reported that plant density alters N allocation. In partitioning, medium planting density results in high leaf N allocation to the P/S apparatus and its different partitioning components (Yao et al., 2015). Recently, Liu et al. (2022) demonstrated that N application significantly affects the N utilization efficiency. N at 180 kg ha^–1^ was applied at the flowering stage, promoting N utilization and efficiency in cotton grown under a wheat-cotton double cropping system (Liu et al., 2022).

In another study, Du et al. (2016) evaluated different cropping systems and reported reduced N accumulation rates and NUE in cotton in wheat-cotton rotations compared with monoculture (Du et al., 2016). Wei et al. (2012) studied the effects of different irrigation methods and N application rates. They reported that a moderate N application rate with drip irrigation promoted N uptake, translocation, and efficiency in cotton grown in arid regions (Wei et al., 2012). N application at 150 kg ha^–1^ and straw incorporation significantly improved N uptake and NUE, as reported by Wang et al. (2020). Niu et al. (2020) examined the responses of various cultivars and N application rates to NUE. They reported that CRI 69 and ZZM 1017, as N-inefficient cultivars, had increased N uptake and translocation into different parts and ultimately NUE in cotton (Niu et al., 2020). Plants inoculated with *Azospirillum brasilense* also show better N uptake, translocation into different tissues, and NUE (Saubidet et al., 2002).

### Nitrogen use efficiency regulating enzymes and genes

Plants have evolved various mechanisms involving enzymes and activating genes to increase NUE (Table 2). However, only a few studies have discussed the key enzymes and genes directly or indirectly involved in enhancing NUE in cotton (Iqbal et al., 2020d). NUE highly depends on N uptake, utilization efficiency, and N assimilation rate (Garnett et al., 2015; Hawkesford, 2017). The involvement of the N-assimilation enzyme glutamine synthetase in promoting NUE through increased N harvest index (Brauer et al., 2011), increased number of grains (Martin et al., 2006), biomass production (Oliveira et al., 2002), and photorespiration (Hoshida et al., 2000) has been well documented in previous reports. Cai et al. (2009) documented that glutamine synthetase significantly promoted N uptake by crop plants (Cai et al., 2009). GS has been reported to enhance biomass production (Chichkova et al., 2001) and seed weight (Yamaya et al., 2002). NR and NiR are important enzymes for N assimilation; their role in enhancing NUE has been well reported in terms of enhancing dry weights (Abiko et al., 2010), nitrate content (Lillo et al., 2003), NiR activity (Crété et al., 1997), and NO_2_^–^ assimilation (Takahashi et al., 2001). The GDH (NADH-GDH) has been reported to play a role in assimilating inorganic N to form glutamate by combining ammonium with 2-oxoglutarate (Fontaine et al., 2012).

**TABLE 2:** Nitrogen use efficiency regulating enzymes and their specific roles in plants.

Enzymes	Roles	References
Plastidic isoenzyme-GS2	Involved in primary N assimilation	Masclaux et al., 2000; Hirel et al., 2001
Cytosolic GS isoenzyme-GS1	Involved in the recycling of organic N	Masclaux et al., 2000; Hirel et al., 2001
Glutamine synthetase	Increased N uptake and photorespiration	Habash et al., 2001
Nitrate reductase and nitrite reductase	NO_2_^–^ assimilation and reduced the nitrate levels	Takahashi et al., 2001; Lillo et al., 2003
GS1.3 isoenzyme	Plays a putative role in controlling the yield under variable N conditions	Martin et al., 2006
Alanine aminotransferase	Increased biomass and grain yield	Good et al., 2007
NADH-GOGAT	Involved in glutamate synthesis to promote the growth of plants; promoted inorganic nitrogen assimilation notably in the roots	Tabuchi et al., 2007; Garnett et al., 2015
Cytosolic asparagine synthetase and carbamoylphosphate synthase enzymes	Increased ammonium assimilation	Lam et al., 2003; Ranjan and Yadav, 2019

It is also well documented that NADH-GDH facilitates ammonium assimilation, which has an advantage over glutamine synthetase. The plastidic isoenzyme (GS2) is also involved in primary N assimilation, and the cytosolic GS isoenzyme (GS1) is involved in the recycling of organic N (Masclaux et al., 2000; Hirel et al., 2001). According to Martin et al. (2006), the GS1.3 isoenzyme plays a putative role in controlling yield under variable N conditions. NADH-GOGAT, a pyridine nucleotide-dependent GOGAT isoenzyme, is present in bundle-sheath cells and is involved in glutamate synthesis to promote plant growth (Tabuchi et al., 2007; Plett et al., 2016). The essential roles of another N-assimilation enzyme, alanine aminotransferase, in increasing biomass and seed yield have been well documented in previous reports (Good et al., 2007; Shrawat et al., 2008). According to Lam et al. (2003) and Ranjan and Yadav (2019), increased ammonium assimilation is also a result of the involvement of carbamoyl phosphate synthase and cytosolic asparagine synthetase enzymes (encoded by ASN1, ASN2, and ASN3).

### Nitrogen use efficiency responsive genes manipulation

Different breeding approaches have been used in recent years to select the most appropriate traits for improving NUE. In plants, NUE is a complex trait that depends on the availability of soil N and various internal and external factors, including photosynthetic carbon fixation. NUE, the ratio of grain yield and N uptake, also varies with N application rates, where different genes are expressed in plants according to N rates (Hirel et al., 2001; Good and Beatty, 2011). According to Hirel et al. (2001), cytosolic GS genes, namely, *gln1, gln2*, and *gln4*, regulate leaf cytosolic enzyme activity. The GS gene *gln4* has been reported as a housekeeping gene that controls NUE and influences yield by promoting ammonia assimilation (Hirel et al., 2001). A previous study reported enhanced NUE and yield upon overexpression of Gln1-3 and Gln1-4 (He et al., 2014). In an investigation by Castaings et al. (2009), it was concluded that the regulatory gene *NLP7* was identified in Arabidopsis to regulate the nitrate content (Castaings et al., 2009). In a recent study, Cao et al. (2017) reported that regulating genes *ZmNLP6* and *ZmNLP8* help in nitrate signaling to increase the productivity of model plants (Cao et al., 2017). Table 3 lists the genes involved in NUE.

**TABLE 3:** Genes directly or indirectly involved in nitrogen use efficiency.

Genes	Direct/Indirect functions	References
*GS2*	Increased the photorespiration and biomass production	Migge et al., 2000
*NiR*	NO_2_^–^ assimilation	Takahashi et al., 2001
*GOGAT*	Improved plant biomass and seed weight	Yamaya et al., 2002
*AtDUR3*	Upregulation of *AtDUR3* plays a putative role in organic N uptake	Liu and Tsay, 2003
*GDHA*	Increased ammonium assimilation, biomass, and dry weight	Mungur et al., 2006
*GS1*	A glutamine synthetase gene had higher grain yield, NUtE, and kernel number upon overexpression	Gallais et al., 2006
*AlaAT*	Increased plant biomass and seed yield	Good et al., 2007
AMT genes (*AMT 1.1*, *1.3*, *1.5*)	Help in ammonium uptake and its transport in plants	Yuan et al., 2007
*HvAlaAT*	As alanine aminotransferase gene increased biomass and grain yield	Shrawat et al., 2008
*AtNRT1.1*	It can result in better nitrate uptake when acting as a nitrate sensor	Ho et al., 2009
*ENOD93–1*	Increased plant biomass and seed weight	Bi et al., 2009
*NIA* (*Nia1* and *Nia2*)	As nitrate reductase genes significantly increased grain weight, dry biomass, and protein contents when overexpressed in model crops	Lillo et al., 2003; Zhao et al., 2013
*DOF1*	Promoted the growth and N uptake	Wang et al., 2013
*AGL21*	Increased plant biomass and seed weight	Yu et al., 2014
*Gln1-3* and *Gln1-4*	Overexpression of these genes improved the yields and enhanced NUE	He et al., 2014
*OsAMT1;1*	As an ammonium transporter gene, helped in enhancing NUE	Ranathunge et al., 2014
*OsAMT1;1GS1*	Facilitate in achieving higher seed yield, NUE, and more numbers of seeds	Ranathunge et al., 2014
*TOND1*	Enhanced tolerance under N deficit condition	Zhang et al., 2015
*OsNRT2.1*	Increased biomass production	Chen et al., 2016
*NRT1.1B* and *OsNRT2.3b*	As nitrate transporter genes helped in increasing the biomass production and seed yield to help in enhancing NUE	Gallais et al., 2006; Hu et al., 2015; Fan et al., 2016b
*OsNRT2.3b* and *NRT1.1B*	Worked as nitrate transporter genes to enhance NUE	Hu et al., 2015; Fan et al., 2016a
*NLP7*, *ZmNLP6*, and *ZmNLP8*	*NLP7* regulates the nitrate content, and *ZmNLP6* and *ZmNLP8* are helped in nitrate signaling to increase the productivity of model plants	Castaings et al., 2009; Cao et al., 2017
*GLN1* and *GLN2*	*GLN2* nuclear gene involved in the assimilation and re-assimilation of ammonium produced by nitrate reduction and from photorespiration, respectively. *GLN1* helped in the recycling of ammonium during leaf senescence.	Bernard and Habash, 2009; Ranjan and Yadav, 2019

The glutamine synthetase gene *GS1* and nitrate transporter genes *OsNRT2.3b* and NRT1.1B (NPF6.3B) have been reported to promote biomass production and seed yield, thereby enhancing NUE (Hu et al., 2015; Fan et al., 2016a). Similarly, according to Zhao et al. (2013), the expression of the nitrate reductase gene NIA promotes seed weight and protein content (Zhao et al., 2013). In another study, Shrawat et al. (2008) demonstrated that the alanine aminotransferase gene *HvAlaAT* significantly increases biomass production and seed yield (Shrawat et al., 2008). In addition, the ammonium transporter gene *OsAMT1;1GS1* has been reported to have a higher seed yield, nitrogen use efficiency, and number of seeds (Ranathunge et al., 2014). In plants, the *GLN2* nuclear gene is involved in the assimilation and re-assimilation of ammonium produced by nitrate reduction and photorespiration (Ranjan and Yadav, 2019). Similarly, according to Bernard and Habash (2009), the *GLN1* gene facilitates the recycling of ammonium during leaf senescence (Bernard and Habash, 2009). The dual affinity transporter protein AtNRT1.1 (*AtNPF6.3*), which is expressed in root tips, can act as a nitrate sensor and improve nitrate uptake (Tsay et al., 2007; Ho et al., 2009), even under low N conditions (Liu and Tsay, 2003). Similarly, Li et al. (2006) demonstrated that *AtNRT2.1* and *AtNRT2.2* are involved in high-affinity nitrate uptake. Improved NUE via increased biomass has been well reported as a result of gene expression, including *GS1* (Fuentes et al., 2001), *GOGAt* (Chichkova et al., 2001), *AlaAT* (Good et al., 2007), and *ENOD93–1* (Bi et al., 2009) (Table 3).

## Integrated approaches to enhance nitrogen use efficiency in cotton

Nitrogen use efficiency is defined as a plant’s ability to absorb nutrients from the soil, assimilate them, and utilize them to maximize crop yield (Hawkesford, 2017; Erisman et al., 2018). Increasing crop production is largely associated with fertilizer use and application rates. The optimum supply of nutrients plays a significant role in increasing crop production and NUE (Erisman et al., 2018; Fernie et al., 2020). In agricultural farming systems, N management should achieve higher crop productivity without polluting the environment (Klerkx et al., 2010). In the form of nitrate, N is a mobile nutrient that severely impacts the environment through GHGs emissions (Hristov et al., 2013). Plants obtain N from different sources, including residual N, organic matter decomposition, and biological N fixation, and it is imperative to understand the contribution of these sources to enhance NUE (Plett et al., 2020). Various approaches have been used in recent years to manage N and enhance the NUE. Soil and plant-based analysis, based on SOM, yield goal, N credit from the previous crop, manure, and irrigation water, is among the most commonly used techniques for N management in different crops (Dobermann and Cassman, 2002). According to a recent study by Guo et al. (2019), soil-based approaches are more suitable for areas with a homogeneous landscape and environmental and soil conditions with similar EC, crop productivity, and resource use efficiency (Benedetti et al., 2019; Guo et al., 2019). Variables such as slopes, soil depth, and drainage within a landscape significantly affected seed yield. Higher N fertility status has been reported in foot slopes due to higher SOM content and flow of water, while it is also well established that soils with upper landscape positions are poor with SOM (Sharma et al., 2017).

### Fertilizer placement and timing

Compared to other nutrients, N is more prone to many soil transformations that occur within the soil, particularly above the soil layer, and can influence NUE (Yadav et al., 2017). Significant N losses have been reported owing to leaching, soil runoff, and volatilization. Therefore, it is necessary to choose appropriate methods of N application to maximize N availability to crop plants and NUE (Sharma et al., 2017). The broadcasting method of N application is not recommended because it causes severe losses in crop yield, and N is applied through volatilization and immobilization (Lasisi et al., 2020; Nasielski et al., 2020).

### Use of tissue analysis for nitrogen management

Sensitive plants have been used for a long time as a marker of soil nutrient status. Some crop species are good markers for overall growing conditions, as they are immediately correlated with climatic conditions and soil management techniques, as discussed recently by Sharma and Bali (2017). In a recent study, Iqbal et al. (2020b) demonstrated that increased availability of N to crop plants results in N accumulation in different tissues, thereby increasing the chlorophyll content and net photosynthetic rates (Iqbal et al., 2020b). A positive correlation between chlorophyll content, corn yield, and N accumulation has been reported previously (Han et al., 2016; Ali et al., 2020).

### Breeding program

Breeding for improved NUE can be attained through an assortment of different components (Cormier et al., 2013); where compensation and regulation are abundant and dependent on N application rates, plant species, and growth stage, directing obstacles to create effective NUE phenotypes (Cormier et al., 2016). However, omics-based studies offer results that enable us to focus on routes for improvement (Cormier et al., 2016). In addition, high-throughput phenotyping using high-throughput genotyping techniques will promote research on variable environments and species, which could significantly enhance their nutrients (Banerjee et al., 2020).

## Conclusion

This review critically assessed various agronomic and molecular approaches for quick and efficient NUE in cotton production. Here, we describe opportunities to increase NUE through new agronomic practices such as 4R’S nutrient stewardship and recently developed molecular strategies, including manipulating metabolic pathways and transport in cotton plants. However, several concerns remain to be addressed. These could improve metabolic activities, the role of nitrogen transporters, signaling and sensing of metabolic pathways, and the relationship between sources and sinks. Ultimately, we recognize that improving NUE in cotton will be equally beneficial for the environment and growers to maximize their profits. This also involves expanding the growers’ objectives from the primary emphasis on cotton production for profit, which is crucial for livelihoods, to adding to the stewardship of the land and surrounding environment, which is essential for sustainability in cotton production. Understanding and following these motives will assist in building better education and other activities to eliminate barriers to nitrogen use efficiency in cotton farming.

## Author contributions

MC, QA, LZ, and LS-L: conceptualization, visualization, and writing—original draft. LZ and LS-L: writing—review and editing, validation, conceptualization, supervision, and funding acquisition. MH, SH, MA, TJ, TM, MS, AU, WA, and SN: writing—review and editing. All authors contributed to the article and approved the submitted version.
